# DNA Damage Repair System in Plants: A Worldwide Research Update

**DOI:** 10.3390/genes8110299

**Published:** 2017-10-30

**Authors:** Estela Gimenez, Francisco Manzano-Agugliaro

**Affiliations:** 1 Central Research Services, University of Almería, C/ Sacramento s/n, Almería 04120, Spain; estela@ual.es; 2 Engineering Department, University of Almería, C/ Sacramento s/n., Almería 04120, Spain

**Keywords:** DNA damage repair, plant, worldwide, bibliometric, Scopus

## Abstract

Living organisms are usually exposed to various DNA damaging agents so the mechanisms to detect and repair diverse DNA lesions have developed in all organisms with the result of maintaining genome integrity. Defects in DNA repair machinery contribute to cancer, certain diseases, and aging. Therefore, conserving the genomic sequence in organisms is key for the perpetuation of life. The machinery of DNA damage repair (DDR) in prokaryotes and eukaryotes is similar. Plants also share mechanisms for DNA repair with animals, although they differ in other important details. Plants have, surprisingly, been less investigated than other living organisms in this context, despite the fact that numerous lethal mutations in animals are viable in plants. In this manuscript, a worldwide bibliometric analysis of DDR systems and DDR research in plants was made. A comparison between both subjects was accomplished. The bibliometric analyses prove that the first study about DDR systems in plants (1987) was published thirteen years later than that for other living organisms (1975). Despite the increase in the number of papers about DDR mechanisms in plants in recent decades, nowadays the number of articles published each year about DDR systems in plants only represents 10% of the total number of articles about DDR. The DDR research field was done by 74 countries while the number of countries involved in the DDR & Plant field is 44. This indicates the great influence that DDR research in the plant field currently has, worldwide. As expected, the percentage of studies published about DDR systems in plants has increased in the subject area of agricultural and biological sciences and has diminished in medicine with respect to DDR studies in other living organisms. In short, bibliometric results highlight the current interest in DDR research in plants among DDR studies and can open new perspectives in the research field of DNA damage repair.

## 1. Introduction

Since their origins, the genomes of all organisms are exposed to the harmful effects of several environmental and metabolic factors. DNA damage can be classified into two types according to its origin: endogenous and exogenous. The endogenous DNA damages mainly come from mistakes included during DNA replication, by errors in chromosome distribution in meiosis or mitosis, or from DNA aging induced by alkylation or by reactive oxygen species derived from cellular metabolism [1]. On the other hand, exogenous DNA damage arises when environmental, physical and chemical agents such as ultraviolet and ionizing radiations, alkylating agents, and crosslinking agents harm the DNA molecules [2]. So as to preserve genomic integrity and to avoid the accumulation of lesions within the genome, molecular mechanisms have evolved to repair the numerous DNA lesions that occur daily on various sites of the DNA, e.g., single and double-stranded DNA breaks, insertion/deletion, base-pair mismatches, base and sugar damage, inter-strand DNA crosslinking, and alkylation lesions [1,3,4]. DNA damage repair (DDR) processes implicate specialized proteins and regulatory pathways that mediate the detection and repair of DNA lesions. Certain repair mechanisms are highly specialized for a specific damage while other pathways operate to repair numerous lesions. Thus, different DNA repair pathways often possess overlapping key roles in the process [4,5]. For instance, the nucleotide and base excision repair pathways repair DNA helix-distorting lesions and single-strand breaks, mismatch repair pathways deal with base mismatches and insertions/deletions, while DNA double strand breaks are corrected by the non-homologous end-joining pathway or with homologous recombination (HR) pathways. In addition, the Fanconi anaemia pathway works together with diverse HR factors to repair inter-strand DNA cross-link lesions. All these pathways are active throughout different stages of the cell cycle, allowing the cells to repair DNA damage [4]. 

The recognition and successive repair of DNA damage not only includes the activation of DNA repair processes but also implies arrest of the cell cycle to allow time for DNA lesions to be resolved before that the cell cycle continues [4,6]. So, cell cycle arrest and the activation of DNA repair mechanisms are initiated as a DNA damage response. DNA damage triggers a response through a signalling cascade composed of sensors, transducers and effectors, which involve complex interactions and post-translational modifications and lead both cell cycle arrest and the activation of DNA repair mechanisms [7]. Then, cells can activate checkpoints at various stages of the cell cycle to avoid advance of the cell cycle, and therefore preventing replication of mismatched DNA templates or segregation of modified chromosomes [8,9,10]. If, on the contrary, the level of damage is too severe and incompatible with DNA repair, cells can decide not to activate the repair mechanism for elimination of DNA damages and proceed toward apoptotic cell death, autophagy or senescence mechanisms, with the aim to eliminate damaged cells or to position them into a non-dividing phenotype [11,12,13] in order to prevent the transmission of pro-mutagenic lesions to daughter cells [6,14,15]. A mistake in this process can stabilize pro-mutagenic lesions during replication and be transmitted to daughter cells during mitotic segregation of the chromosomes [15,16]. So, the repair processes are key to maintaining genome stability and integrity in cells and continuing replication and transcription [14], and are responsible for monitoring genome health [15]. Given that the integrity of the genomes has always been exposed to the impact of exogenous and endogenous agents, evolution has provided organisms with numerous DNA repair pathways. These DNA repair mechanisms not only ensure the defence of the genome against damage but also assure the transmission of genetic information through the generations [2,11].

DNA repair mechanisms imply a set of enzymatic activities such as nucleases, recombinases, polymerases, topoisomerases, kinases, ligases, glycosylases, helicases, phosphatases and demethylases, which repair DNA by chemical modifications [17]. These activities must be finely regulated because the integrity of DNA will be compromised if some of enzymatic activities are employed wrong or if access DNA is allowed at the incorrect time or genomic site. Therefore, eukaryotic cells developed approaches to activate the right enzymatic factors in the right genomic site at the right time [4,9]. Not surprisingly, when DNA damage repair pathways are disrupted or deregulated, mutagenesis and genomic instability increases, promoting cancer development [18,19,20]. Other sicknesses, such as neurodegenerative disorders, result also from diverse alterations happened in these repair processes. Likewise, many aging-related diseases are attributed to shortening of chromosomal ends and alterations in the adequate combination of DNA repair pathways. Despite the fact that DNA damage repair deficiencies are related with a wide range of human pathologies, research field on defects in DNA damage repair mechanism has been focused on cancer [2,21]. Actually, it has been proved that hereditary mutations of diverse genes that regulate DNA repair processes cause or contribute to cancer development. However, there is strong and increasing evidence that defects in DNA repair mechanisms contribute more broadly to sporadic cancers. In any case, these data highlight the importance of DNA damage repair mechanisms in human health.

During evolution, plants have also developed complex signalling pathways that mediate DNA damage, recognizing and activating of repair mechanisms, as well as different DNA repair pathways [22]. With some exceptions [22,23], plants share common DNA repair mechanisms, which have been previously described in the other eukaryotic systems such as yeast and mammals [24]. Similar to animals, some of the repair mechanisms are highly focused on a specific damage (photoreactivation), while other repair pathways such as excision or recombination are responsible for correction of a variety of lesions [25]. In plants, the impairment of DNA damage repair processes also changes cellular activity and modifies physiological processes such as the cell cycle, transcription and protein synthesis, which eventually alter the normal development and growth of the whole organism [26,27,28]. 

As sessile organisms, plants are especially susceptible to the DNA harmful factors present in the air, soil, and water. Hence, the development of detection and repair mechanisms of DNA damage are essential to ensure genomic integrity and stability through the correction of DNA lesions in order to recover the original genetic information [29,30]. However, DNA repair pathways are not mistake-free, being able to cause potential mutational alterations that may be inheritable to daughter cells. These kinds of errors in some DNA repair mechanisms raise the genetic variability and diversity of the populations, which contributes to the evolution of plant genomes [31]. Currently, intensive research is being carried out on Arabidopsis and rice, which has enormously improved the current knowledge on the molecular mechanisms that regulate the DNA damage detection and repair pathways in plants. The conservation of DNA damage repair processes among species will not only facilitate the characterization of DNA repair pathways of other plant model and crop species but will also enable the implementation of interdisciplinary studies that traverse the typical limits found between animal and plant biology. 

During recent decades, the development of Scientometrics [32,33], Informetrics [34], and Bibliometrics [35,36] has allowed the study of scientific trends in specific fields. In turn, these analysis techniques of scientific production are necessary for the assessment of the current state of research as well as the contributions of researchers and countries in the fields of knowledge, which will guide the future lines of research towards specific fields [35]. Furthermore, the use of bibliometric indicators to measure the results of the scientific production in a specific field by a country or organization can be considered also as the economic and social indicators since they are investing huge resources in it [37]. So, the aim of this work is to perform a descriptive and retrospective bibliometric study on the worldwide of scientific production in the field of DNA damage repair and its relationship with plant research.

## 2. Materials and Methods 

In this study, all the publications were extracted from Elsevier Scopus database. A comparative analysis related to the journal coverage of Web of Science and Scopus shows that the journals in Web of Science (WoS) are lower than in Scopus [38]. Furthermore, the correlations between both databases for the number of papers and the number of citations are extremely high (*R*^2^ ≈ 0.99) [39]. The advantages of Scopus are shown in several research papers and therefore also used for numerous bibliometric analysis [37,40]. Other databases such as PubMed are very useful in several scientific fields, but do not allow massive information downloads and are therefore not useful for bibliometric studies [41].

The search was conducted in September 2017 to collect academic publications containing the terms “DNA Damage Repair” or “DNA Damage Repair AND Plant” in the title, abstract and/or keywords. The following search queries were used: (TITLE-ABS-KEY({DNA Damage Repair}), and another one specific for plant: (TITLE-ABS-KEY({DNA Damage Repair}AND{plant}), and limited to period 1970 to 2016. The publications obtained were assessed and classified based on the following aspects: number of publications per year, type of document, distribution by subject categories and by journals, as well as distribution by institution and by country. The records obtained were conveniently processed using spreadsheets and generating the corresponding graphs that allow easier visualization of the results. 

As a key method for content analysis, word frequency analysis has been widely performed to indicate the core content and research object of academic literature. The Wordart, a kind of word cloud software, was used to visualize the importance of words in the text (https://wordart.com/). In this study, keywords most present in the articles of DDR and DDR & Plant research fields published in the last decade (2006 to 2016) were uploaded into the Wordart software. Previously, keywords such as “article,” “review” or “DNA damage repair” were removed, since that they did not provide information to this study. Additionally, if duplicated keywords like “human” and “humans” were observed, then, it was proceeded to merge them as one keyword. Finally, keywords most present in the articles of DDR and DDR & Plant research fields were graphically represented. The frequency of word occurrence was showed by the size of font. 

Citation amount is a powerful and popular analytical tool that allows for studying the relative impact that a publication has on the scientific community. To analyse the impact of publications related to DDR and DDR & Plant, *h*-index indicator was created in this field. *H*-index, defined as h of one's total articles has at least h citations each [42], can study both the quantity (number of publications) and quality (number of citations) of a journal, a scientist, an institution and even a country [43,44]. A higher value of *h*-index generally indicates a greater scientific attainment. These methodologies were used successfully in other bibliometric studies [36,45]. 

## 3. Results and Discussion

### 3.1. Evolution of Scientific Output

**Figure 1:**
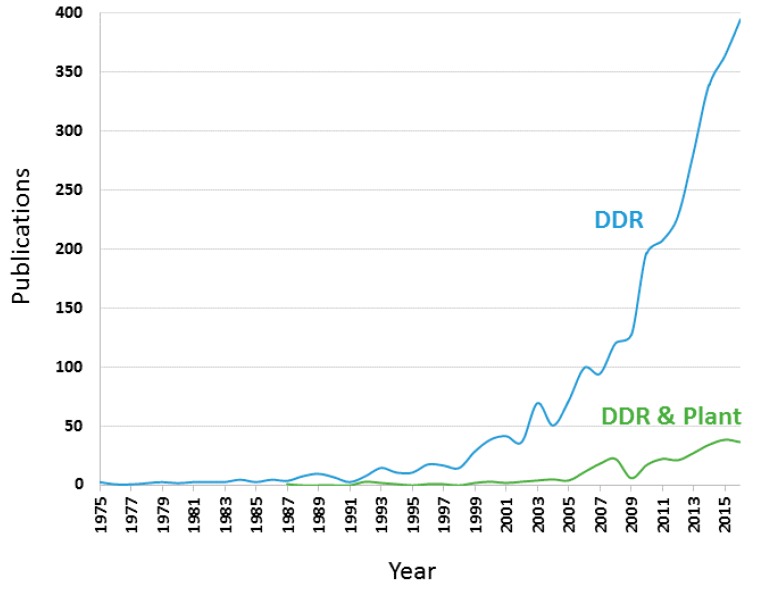
Trends in publications in the DNA damage repair (DDR) research field and the DDR research in plant field from 1975–2016.

Scientific outputs of the “DNA damage repair” research field experienced a substantial growth of publications. A total of 2921 documents with DDR term in titles, abstracts or key words were retrieved. The evolution in the period 1970–2016 of the documents analysed in the topic of DDR showed that no article about DDR appears until the year 1975, and few documents on DDR were published for first years. More than 10 documents per year were published from 1993 and since then a progressive increase of DDR related publications has happened until reaching a maximum of 395 documents published in 2016. During this period, years 2010, 2013 and 2014 highlighted by their higher number of publications with respect to more constant growth in the period 1970–2016 (Figure 1). If the scientific outputs of “DNA damage repair” are exclusively considered to be in the plant field, only 284 documents are recovered, which shows that plants are only contemplated in around 10% of DDR related studies. This low percentage is likely since no article about DDR in the plant field appears until the year 1987, and none was published in years 1988, 1989, 1990, 1991, 1995 and 1998. Ten publications per year were recently reached in the year 2006, and since then a slow and continuous rise of publications has happened until reaching a maximum of 38 documents published in 2016. From the year 2003, there was a prominent increase in publications on DDR research as a whole—this fact could also produce the increase of the publications in the plants field observed in 2006. DDR-related documents in the plants field account for 10% of total DDR publications from the year 2010 to the present (Figure 1), suggesting that the increase of documents in the DDR research field in plants is comparable to rise of total DDR documents. It indicates that the interest of scientific community on DDR research in the plant field is beginning, and suggests that a constant growth in the number of DDR publications in plants is expected in the coming years. 

It should be noted that these data are the results of the search in the database of Scopus. If this search is made in another database, e.g., PubMed, the results may change, but not significantly to alter the conclusions performed in this analysis.

### 3.2. Types of Publications

Documents in the DDR field can be categorized into 11 types. The most frequently used document type was “Article” which accounted for 77.98% (2278 records) of total publications, followed by “Review” with 16.05% (469 records), “Conference paper” with a merely 2.01% (59 records), “Book Chapter” with 1.88% (55 records) and “Short Survey” with 0.89% (26 records). The records of other types such as “Note,” “Erratum,” “Article in Press,” “Book,” “Editorial” and “Letter” accounted for 1.16% (Table 1). However, if the word “Plant” was included together with DDR term in titles, abstract or key words, documents recovered can be only classified into 7 types since terms DDR and plant together were not found in publications type “Letter,” “Note,” “Editorial” and “Book.” The most frequently used document type with both words was again “Article” with 217 records, which accounted for 76.41% of total DDR & Plant publications, followed by “Review” (48 records), “Book Chapter” (8 records), “Short Survey” (5 records), “Conference paper” (4 records), “Article in Press” (1 records), and “Erratum” (1 record) (Table 1). These results indicate that most of authors prefer mainly to publish their important findings in article and review formats. The fact that articles and reviews represent about 90% of all document types implies that if the number of articles and reviews of DDR & Plant account for 10% of global DDR articles and reviews, all DDR & Plant related documents also will account for 10% of total DDR publications as mentioned above.

### 3.3. Distribution of Output in Subject Categories and Journals 

**Figure 2:**
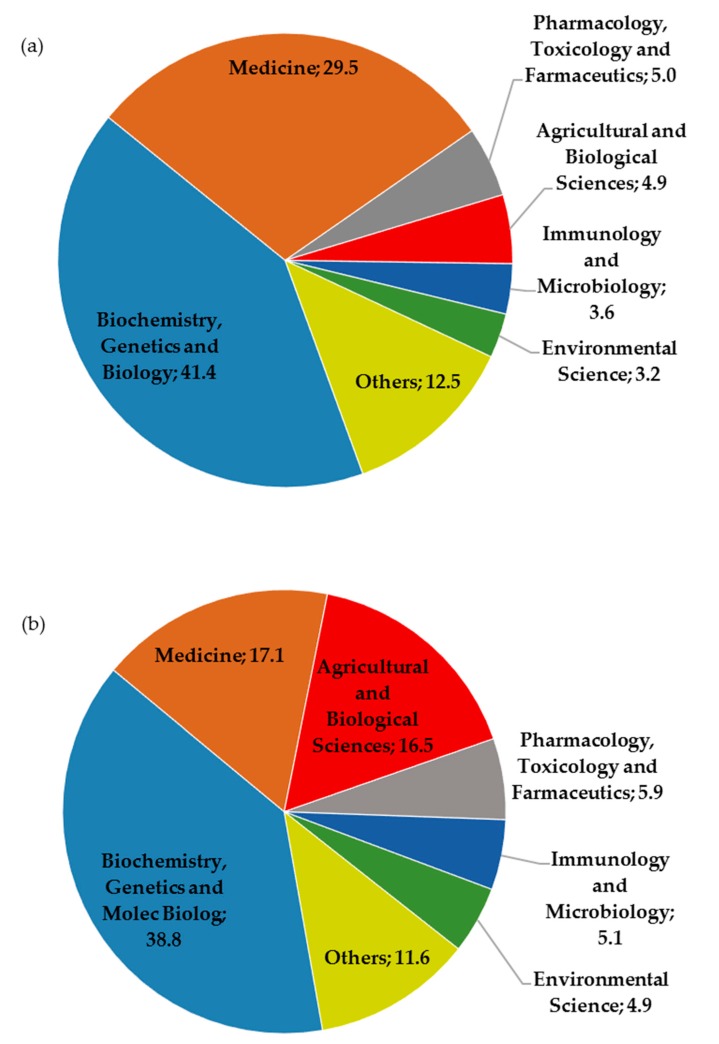
Distribution (%) of worldwide research on: (**a**) DDR and (**b**) DDR & Plant by subject area, as classified by Scopus.

Based on the Scopus classification, the distribution of publications on DDR research fields covered a total of 24 subject areas. The largest number of documents corresponds to biochemistry, genetics and molecular biology (2062 records, 41.40%), while the second area in terms of number of publications is medicine (1469 records, 29.49%). The third area is pharmacology, toxicology and pharmaceutics (249 records, 5.00%), the fourth is agricultural and biological sciences (244 records, 4.90%), the fifth Immunology and microbiology (178 records, 3.57%) and the sixth area is environmental sciences (158 records, 3.17%). Among these six areas they account for about 90% of all publications (Figure 2). It should be noted that a document can be assigned to more than one area at the same time. The distribution of publications on DDR in the plant research field reduced the number the subject areas, enclosing only 18 subject areas. Six first areas were the same that those showed by global DDR research field i.e., biochemistry, genetics and molecular biology is the first area according to number of publications (197 records, 38.78%), medicine is the second area (87 records, 17.13%), while that the agricultural and biological sciences area reached the third position with 84 papers, which accounted for 16.54%. So, the pharmacology, toxicology and pharmaceutics area (5.91%) got relegated to the fourth position, while immunology and microbiology (5.12%) and environmental sciences (4.92%) areas maintained the fifth and sixth positions, respectively (Figure 2). It is important to note that representation of the agricultural and biological sciences area increased from a 4.9% to a 16.54% when DDR studies were developed in plants. The increase of 10% in the agricultural and biological Sciences area implied a reduction about 10% in medicine area, while the participation of other areas was not altered. DDR study is mainly focusing on aging, cancer and other diseases, which entails a high number of DDR publications in medicine area. However, when DDR projects are focused on plants, despite that said projects can be approached to cancer research, results obtained are mainly published in the agricultural and biological sciences in detriment of medicine area.

Regarding to journals, all the 2921 papers of the DDR field referred to 159 different journals. Despite low number of publications by journal, the high number of journals in which authors published their DDR research results indicated the breadth of publication distribution as well as the broad interest in DDR research from various perspectives. Table 2 lists the top 10 journals, including only those with more than 30 publications, in which DDR results have been published. A total of 468 papers were included in the first ten journals, representing only 16% of the total. This low percentage was due to the fact that there were numerous journals with a low number of publications of the DDR field (114 journals published less than 10 documents). Heading the list of items published was *PLoS ONE*, which is a powerful multidisciplinary journal even though this journal emerged in the year 2006. Other multidisciplinary journal such as *Proceedings of the National Academy of Sciences of The United States of America* (*PNAS*) was also in top 10 journals, in the last position. However, most of authors preferred to publish their important findings in professional journals on cancer or on DNA. Regarding cancer journals, *Cancer Research* was the second most productive journal with 66 publications, while *Oncogene*, *Oncotarget* and *Clinical Cancer Research* occupied the fourth, seventh and ninth positions respectively (Table 2). In the specific journals of nucleid acids, *DNA repair*, *Cell Cycle* and *Nucleid Acids Research* occupied the fifth, sixth and eighth positions in the top ten journals respectively (Table 2). Respect to 284 documents of DDR research field in plants, they were published in 152 different journals, of which 115 only published one document about DDR & Plant research, other 19 journals only two publications and 7 journals published 3 reports. Similar to DDR research field, despite low number of publications on DDR & Plant by journal, the high number of journals in which authors published their results indicated the broad interest in DDR & Plant research from numerous perspectives. Table 2 also lists the top 10 journals in DDR & Plant research. Compared with the top 10 journals in which global DDR results were published, professional journals on cancer such as *Oncotarget*, *Clinical Cancer Research* and *Cancer Research* got relegated from the top 10 journals in DDR & Plant research, in a similar way as happened with specific journals *Cell Cycle* and *Journal of Biological Chemistry*. As expected, four specific plant journals, *Plant Physiology* (1^st^), *Plant Cell* (2^nd^), *Frontiers in Plant Science* (4^th^) and *Plant Journal* (5^th^) headed top 10 journals. Plant-related research results are usually published in plant specialized journals. So, documents about DDR in plant are published in journals specific of plants such as *Plant Physiology, Plant Cell, Frontiers in Plant Science* and *Plant Journal*. These journals are habitually included in the subject area agricultural and biological sciences, which cause an increase in the number of publications in the agricultural and biological sciences area as above mentioned. 

As to the *h*-index values of top ten journals in terms of number of publications on DDR research, *Cancer Research* (34), *Oncogene* (28), *PNAS* (27) and *Journal of Biological Chemistry* (25) had the largest *h*-index despite the fact of that these journals did not head top ten journals (Table 2). *PLoS ONE* journal that headed top ten journals in terms of number of publications had an *h*-index 20, together with *DNA Repair* journal. Finally, *Clinical Cancer Research, Nucleic Acids Research, Cell Cycle* and *Oncotarget* journals had an *h*-index 19, 17, 16 and 12, respectively. These results suggested that a journal can publish a high number of DDR related documents although they have low impact on scientific community, as happened with *PLoS ONE* journal. However, *PLoS ONE* has emerged recently, which could be the cause of the low *h*-index showed. On the contrary, *PNAS* journal published few documents on DDR research but the *h*-index of this journal in this subject was very high (Table 2), suggesting that the impact of DDR-related publications in the *PNAS* journal was very high. In contrast, *h*-index values of top ten journals in terms of number of publications on DDR & Plant research agreed with the number of documents published by journal. That is, when number of DDR & Plant-related documents published in a journal was high, the *h*-index in DDR & Plant research of the journal was also high (Table 2). Thus, *Plant Physiology* journal with highest number of DDR & Plant publications (9 records) had the highest *h*-index value (7) (Table 2). 

### 3.4. Publication Distribution by Countries and Institutions

The DDR research field has been developed in more than 160 institutions. Table 3 shows the 20 most productive institutions, with more than 25 publications on DDR processes in the period studied. The first four institutions are from USA, it is not until the fifth place in the rankings that a non-US institution can be found, that being the Chinese Academy of Sciences, which is followed by another Chinese institution, the Ministry of Education China. In fact, both organizations are, together with the Inserm (France) and the University of Toronto (Canada), the only non-USA institutions in the top ten. The 20 most productive institutions are located within six different countries—USA, China, France, the Netherlands, Canada and UK (Table 3). Contrary to what happens in institutes implicated in global DDR research, a US institution does not appear until the fifth position in the ranking of institutions most productive in the DDR & Plant research field. The first two institutions are Chinese: the Chinese Academy of Sciences and Ministry of Education China; followed by two French organizations: Inserm and Centre National de la Recherche Scientifique (CNRS) (Table 3). Also, the 20 most productive institutions in the DDR & Plant research field are distributed across eight different countries (USA, China, France, Spain, Germany, India, Taiwan and Argentina) instead of six countries where the 20 most productive institutions in global DDR research are located (Table 3). The fact that institutes involved in DDR & Plant research are placed in a higher number of countries suggests that the international scientific community now highlights the interest in DDR research in the plant field. 

Thirteen of the twenty most productive institutions in the DDR field are institutes mainly involved in medicine, health or cancer research fields. As expected, most of these medical institutions did not publish research studies about DDR in the plant field—only five medical institutions collaborated in DDR & Plant studies (Table 3). This five institutes are included among 20 organizations more productive in the DDR field in plants, and only Howard Hughes Medical Institute (USA) and the Institut national de la santé et de la recherche médicale, Inserm (France) are represented in the 20 most productive institutions both in global DDR research field and in DDR research in plant field (Table 3). Also, two of twenty most productive institutions in the DDR & Plant field, Centro de Estudios Fotosintéticos Y Bioquímicos, (Rosario) and Max Planck Institut für Züchtungsforschung (Cologne), are exclusively dedicated to plant research. These results suggest again a rise of the implication of the agriculture area in DDR research in the plant field, to the detriment of the Medicine area.

**Figure 3:**
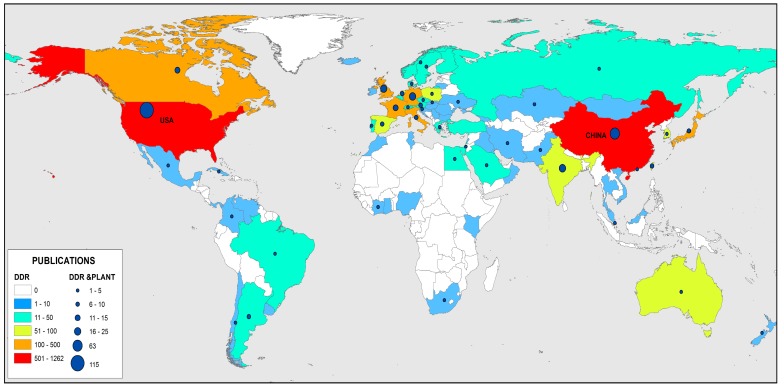
Worldwide research on DDR and DDR & Plant during the period 1970–2016.

As expected according to previous results, the countries with more publications in the DDR research field are USA and China, between them they published 1765 records, which accounted for 60% of total DDR publications (Figure 3). However, despite the fact that the first institutions that were more productive in the DDR & Plant research field are Chinese and French, the countries with more publications about DDR & Plant research are USA and China (Figure 3). This result is likely due to the number of institutions implicated in DDR & Plant in USA is higher than number of organizations occupied in DDR & Plant in France. In addition to USA and China, 72 countries have contributed to the DDR research field while the number of countries involved in the DDR & Plant field is 44 (Figure 3). So, countries involved in DDR & Plant research constitute more than half of those countries implicated in the global DDR research field, despite the fact that the number of publications about DDR in the plant field only accounted for 10% of total DDR publications. This indicates once again the great influence that DDR research in the plant field currently has worldwide. However, the number of languages used in the scientific dissemination of DDR research field is higher than the number used for DDR research in the plant field (Table 4). Research studies about DDR have been published in 15 different languages while those documents about DDR research in the plant field have been only published in three languages, which coincide with the three languages most used in the DDR field (Table 4). As expected, English is the most used language in which to publish both DDR and DDR & Plant documents since English is the international language of science and technology. English is followed by Chinese given that China is the country where the second most DDR and DDR & Plant documents are published. It should be noted that Russian is the third language most used in which to publish DDR and DDR & Plant publications in spite of the low number of DDR and DDR & Plant related documents published by this country. Considering the high impact that DDR & Plant research has on the scientific community, the fact that only three languages have been used in which to publish documents about DDR & Plant is likely due to lacking publications in the DDR & Plant research field currently. However, the ongoing rise in the number of documents about DDR research in plant field suggests that the number of languages employed to publish DDR & Plant related documents will increase in the coming years.

### 3.5. Analysis of Keywords

**Figure 4:**
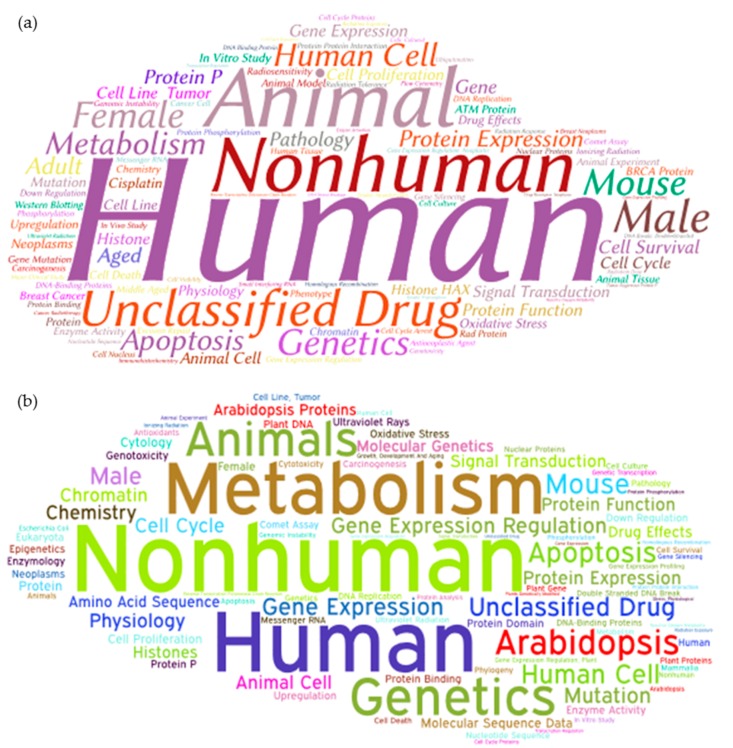
Cloud word based on the main keywords related to worldwide DDR (**a**) and DDR & Plant (**b**) research.

Keywords of an article determine the main subject developed in the research project performed. The study of the keywords in scientific publications permits to identify the trends of the research projects that are developed in a specific research field [46]. Therefore, in this manuscript the keywords of the articles published in DDR research field were analysed. As was mentioned above, keywords included in the search query such as “DNA” orrepair” were not taken into account in order not to distort the results, since they are part of the search term. As can be seen, the words “human” (1^st^) and “nonhuman” (2^nd^) are the most represented in DDR research field, followed of generic words such as “Animals,” “Human Cell,” “Female,” “Male” and “Mouse.” Additionally, numerous specific words related with genetic such as “gene,” “genetics,” “protein expression,” “gene expression,” “histone,” “mutation” and “chromatin” are represented among keywords. Similarly, characteristic words associated with cancer, such as “apoptosis,” “tumour,” “breast cancer,” “carcinogenesis” and “cisplatin,” are also included among keywords of DDR field (Figure 4a). In the DDR research field in plants, the words “human” and “nonhuman” also head the list of keywords more used, but in an opposite way to the global DDR field. “Nonhuman” is the most represented keyword, while “human” is the second keyword more representative. In addition, same generic words and keywords related with genetic and cancer continue in the keyword list in the DDR & Plant research field. However, plants do not get cancer although they constantly expose to ionizing radiation of ultraviolet light [47,48]. So, the use of cancer-related keywords in DDR & Plant publications is due by the fact that the study of DDR mechanism in plants is not only focused on understanding the regulatory genetic pathway that controls DDR process but also expects to help us to better handle human cancers as previous projects proved [49,50]. In addition, new keywords such as “Arabidopsis,” “Arabidopsis protein,” “Plant DNA” and “Plant gene” appear in the DDR publications in plants (Figure 4b). It should be noted that the appearance of “Arabidopsis” word in publications related with DDR research in plants is due to importance of this vegetal species in the plant scientific community. Arabidopsis has a small genome, a short life cycle and various transformation methods available, which make these species an attractive model for the study of DNA damage repair mechanisms and other genetic processes. Even though names of other vegetal species are not included in the keywords of DDR & Plant publications, the DDR mechanism has been studied in other different species to Arabidopsis such as tomato [51,52], rice [53], barley [54] as well as other important crop plants (review in [55]). Additionally, it should be note that since few years the term “Genome Stability” is used in several scientific conferences related to DDR, this is due that DDR is a biological process which helps to “Genome Stability.” This study is focused on DDR, where the associated keyword found is “Genomic instability,” which appears 184 times related to DDR, and 13 times in DDR &Plants.

**Figure 5:**
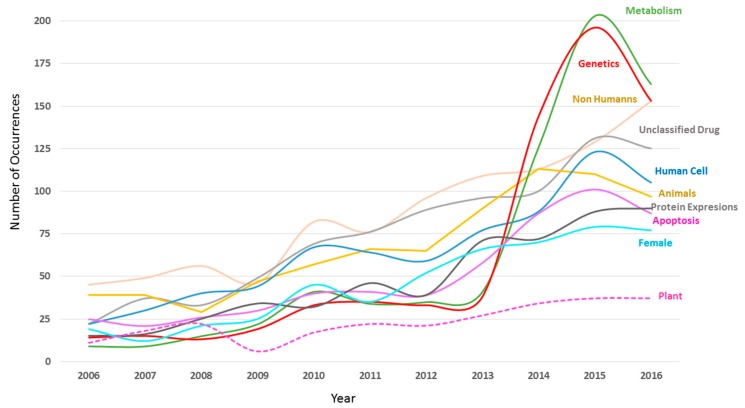
Top ten keywords and Plant keyword evolution related to worldwide DDR research (2006–2016).

Studying the evolution of the 10 keywords more used in DDR-related publications in the period 2006–2016, it is observed as “Animals” keyword emphasize on the rest during period analysed. “Animals” term had a constant growth until year 2013, when highlighted by its quicker rise. Similarly, it should be noted that “Metabolism” and “Genetics” keywords showed a preeminent evolution from 2013. The rest of the keywords maintained constant progress during complete period (Figure 5). Despite “Plant” is not a main keyword in DDR-related publications, we analysed and compared the evolution of the “Plant” word. As expected, results showed that “Plant” term was less contemplated in DDR-related publications than the main keywords during period 2006–2016. However, “Plant” word had a constant growth even in the year 2016, when some main keywords such as “Human,” “Human Cell,” “Animals,” “Genetic,” “Metabolism” and “Apoptosis” presented a decrease (Figure 5). The only keywords, together with “Plant” term, that showed an increase in the number of publications in the year 2016 were “Non-human” and “Protein expression.” These results suggest newly that the interest of scientific community on DDR research in the plant field is starting and is going to continue increasing in the next years.

**Figure 6:**
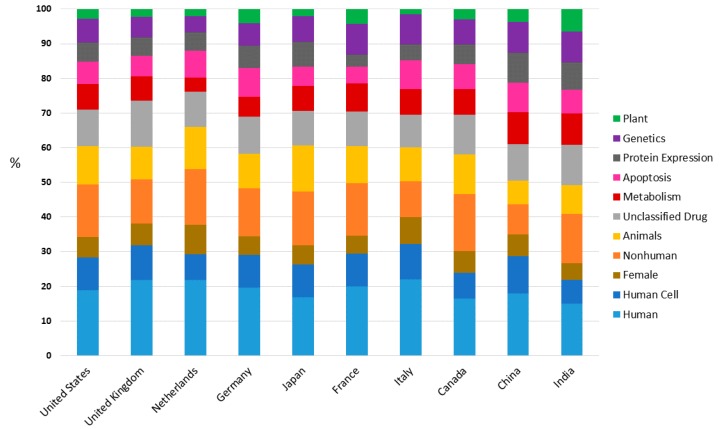
Distribution of main keywords by country as percentage of their own publications.

This study of keywords would not be complete without seeing the relative importance of the keywords for each country in their publications about DDR. Figure 6 shows the percentage that occupies each one of the 10 main keywords by country. Results did not show important differences in the percentages of keywords in different countries. United Kingdom highlighted by using “Unclassified Drugs” term (for instance [56]) more times than any other country. It should also be noted that “Animals,” “Human” and “Non-human” keywords are preferably utilized in Japan, Italy and Canada respectively [57,58,59], while China highlighted by using “Human Cell,” “Metabolism,” “Genetics,” “Apoptosis,” “Protein Expression” keywords more than any other country and in detriment to “Animals” and “Non-human” terms [60,61]. As is the case of Figure 5, the contribution of the “Plant” word in the diverse countries was analysed. Results indicated that India is the country that included more times in their DDR publications the “Plant” word (for instance [62]), which was done in detriment of “Human,” “Human Cell,” “Female,” “Non-human” and “Animals” keywords. These five words accounted for more than 50% of total keywords in all countries except to India (Figure 6). Higher using of “Plant” keywords in DDR-related publications in India is likely since India is found in the top ten of agricultural producers worldwide of main grain crops and its food grain production has increased almost 2 times in the past two decades [63]. In addition, according to FAO, it is estimated in *The State of Food Security and Nutrition in the World* (2017) [63] reports that 190.7 million people are undernourished in India. These data might explain the interest showed by the Indian scientific community in the “Plant” field.

## 4. Conclusions

DNA damage repair mechanisms awaken a great interest in the scientific community, mostly due to the involvement of this process in cancer. Cancer is a main leading cause of death in the 21^st^ century and so all regulatory processes implicated in cancer, including DDR processes, promote interest. During evolution, DNA repair processes have been developed in all organisms, bacterial, fungal, animals and even in plants. Our bibliometric results have showed that interest in the DDR mechanism began more than three decades ago and that said mechanism has been broadly studied—mainly in animals—while the description of these processes in plants was only initiated more recently, at the end of the eighties. Consequently, knowledge about, and research into, the DDR process in plant genomes is still in its early stages. However, recent studies have proved that in spite of the conservation of DNA repair mechanisms in plants, new plant-specific characteristics have been revealed. Big efforts are therefore currently being carried out by the worldwide scientific community to better understand DDR mechanisms in plants. In addition, DDR studies in plants may lead us to better understand why cancers do not affect plants but do affect animals and may also help us to better handle human cancers. Therefore, bibliometric results highlight the interest in DDR research focused on plants and can open up new perspectives on the research field of DNA Damage Repair. 

## Figures and Tables

**Table 1:** Distribution of document types for research on DDR and DDR & Plant.

Document Type	DDR (Records)	DDR (%)	DDR & Plant (Records)	DDR & Plant (%)
Article	2278	77.99	217	76.41
Review	469	16.06	48	16.90
Conference Paper	59	2.02	4	1.41
Book Chapter	55	1.88	8	2.82
Short Survey	26	0.89	5	1.76
Note	13	0.45	~	~
Erratum	7	0.24	1	0.35
Article in Press	6	0.21	1	0.35
Book	4	0.14	~	~
Editorial	2	0.07	~	~
Letter	2	0.07	~	~
Total	2921		284	

**Table 2:** Distribution of publications by source.

	DDR			DDR & Plant		
Position	Source Name	Records	*h*-Index	Source Name	Records	*h*-Index
1	*PLoS ONE*	82	20	*Plant Physiology*	9	7
2	*Cancer Research*	64	34	*Plant Cell*	7	7
3	*Journal of Biological Chemistry*	49	25	*PNAS*	6	6
4	*Oncogene*	48	28	*Frontiers in Plant Science*	5	5
5	*DNA Repair*	43	20	*Plant Journal*	5	4
6	*Cell Cycle*	41	16	*DNA Repair*	4	3
7	*Oncotarget*	40	12	*International Journal of Molecular Sciences*	4	4
8	*Nucleic Acids Research*	35	17	*Mutation Research Reviews in Mutation Research*	4	4
9	*Clinical Cancer Research*	33	19	*Nucleic Acids Research*	4	4
10	*PNAS*	33	27	*Oncogene*	4	4
				*PLoS ONE*	4	3

**Table 3:** Rankings of the 20 most productive institutions in the DDR research field and in DDR research in the plant field.

	DDR			DDR & Plant		
	Affiliation	Country	Records	Affiliation	Country	Records
1	National Institutes of Health, Bethesda,	USA *	82	Chinese Academy of Sciences,	China	13
2	University of Texas M. D. Anderson Cancer Center,	USA *	82	Ministry of Education,	China	9
3	National Cancer Institute, Bethesda,	USA *	64	Inserm, Institut national de la santé et de la recherche médicale,	France *	6
4	Harvard Medical School,	USA *	51	Centre National de la Recherche Scientifique (CNRS),	France	6
5	Chinese Academy of Sciences,	China	46	Howard Hughes Medical Institute,	USA *	6
6	Ministry of Education China,	China	39	UC Davis,	USA	5
7	Howard Hughes Medical Institute	USA *	34	Cincinnati Children’s Hospital Medical Center,	USA *	4
8	Inserm, Institut national de la santé et de la recherche médicale,	France *	31	University of Missouri-Columbia,	USA	4
9	University of Toronto,	Canada	31	Consejo Superior de Investigaciones Científicas,	Spain	4
10	University of Michigan Medical School,	USA *	31	Bose Institute,	India	4
11	Dana-Farber Cancer Institute, Boston,	USA *	30	University of Colorado Health Sciences Center,	USA *	4
12	UT Southwestern Medical Center, Dallas,	USA *	29	Sichuan University,	China	4
13	Erasmus University Medical Center, Roterdam,	The Netherlands *	29	Academia Sinica,	Taiwan	4
14	CNRS Centre National de la Recherche Scientifique,	France	28	University of Texas Health Science Center at San Antonio,	USA *	4
15	Memorial Sloan-Kettering Cancer Center, Philadelphia,	USA *	28	Alfred-Wegener-Institut Helmholtz-Zentrum für Polar- und Meeresforschung,	Germany	4
16	Fudan University, Shanghai,	China	28	UC Berkeley,	USA	4
17	Cancer Research,	UK*	28	Universidad Nacional de Rosario,	Argentina	4
18	University of Oxford,	UK	28	Centro de Estudios Fotosintéticos Y Bioquímicos, Rosario,	Argentina #	4
19	VA Medical Center,	USA *	27	University of California Los Angeles,	USA	4
20	University of California San Francisco,	USA	27	Max Planck Institute für Züchtungsforschung, Cologne,	Germany #	4

* Research Institutions focus on Health, Medicine and/or Cancer; # Plant research centers.

**Table 4:** Languages of scientific output in the DDR research field and DDR research in the plant field.

	DDR		DDR & Plant	
Language	Reports	%	Reports	%
English	2776	94.84	273	96.13
Chinese	106	3.62	8	2.82
Russian	12	0.41	3	1.06
Polish	9	0.31		
Japanese	8	0.27		
French	4	0.14		
German	2	0.07		
Spanish	2	0.07		
Turkish	2	0.07		
Bulgarian	1	0.03		
Dutch	1	0.03		
Italian	1	0.03		
Portuguese	1	0.03		
Serbian	1	0.03		
Ukrainian	1	0.03		
